# UNDERSTANDING MEDICARE-RELATED DECISION-MAKING BY OLDER ADULTS: ROLE OF TECHNOLOGY SUPPORT

**DOI:** 10.1093/geroni/igae098.1539

**Published:** 2024-12-31

**Authors:** Hye Soo Lee, Raksha Mudar, Wendy Rogers

**Affiliations:** University of Illinois at Urbana-Champaign, Champaign, Illinois, United States; University of Illinois at Urbana-Champaign, Champaign, Illinois, United States; University of Illinois at Urbana-Champaign, Champaign, Illinois, United States

## Abstract

Decisions related to Medicare carry significant implications for public health, given older adults’ reliance on this federal program. Our literature review on Medicare decision-making and technology utilization aimed to investigate how technology could be enhanced to better support older adults. We identified pertinent studies by using combinations of search keywords such as “Medicare”, “decision”, and “older adults”, and by using the information from the collected studies. The findings revealed varying levels of Medicare knowledge among older adults, predominantly acquired through human assistance. Government-provided technology for Medicare was either underutilized or perceived as challenging. The evidence indicated that training in decision-making and technology use could prove advantageous. Factors like stress avoidance and the need for reassurance played roles in the decision-making process and plan selections. Considering older adults’ characteristics and preferences in decision-making and technology utilization is crucial for future technological developments, supported by policy and research initiatives.

